# Radiological Signs in Traumatic Cervical Facet Joint Dislocations

**DOI:** 10.5334/jbsr.2314

**Published:** 2021-02-16

**Authors:** Natalie Leong, Ernest Lim, Chi Long Ho

**Affiliations:** 1 National University of Singapore, SG; 2 Sengkang General Hospital, SG; 3 Duke NUS Medical School, SG

**Keywords:** bow-tie sign, headphone sign, laminar space sign, reversed hamburger sign

## Abstract

Unilateral cervical facet joint dislocation (UCFJD) is the most frequently missed cervical spine injury on plain radiographs. If left untreated, UCFJD can progress to bilateral cervical facet joint dislocation. Given the complexity of cervical facet joint dislocations, radiologists rely on metaphorical signs to identify them on radiographs. The “Bow-tie” and “laminar space” signs represent UCFJD on plain radiographs. The “reversed hamburger”, “naked facet” and “headphones” signs represent cervical facet joint dislocations on axial cross-sectional imaging. Illustrating these signs in an engaging manner facilitates pattern-based recognition, which can benefit trainees and radiologists. Moreover, pattern-based recognition can be applied to machine learning.

## Introduction

In the normal anatomical relationship between the cervical facet joints, the inferior articulating facet of the superior vertebral body lies posterior to the superior facet of the inferior vertebral body. This anatomical relationship can be upset in a cervical facet joint dislocation.

Unilateral cervical facet joint dislocation (UCFJD) is one of the most commonly missed cervical spine injuries on plain radiographs [1,2,3], and it can have dire consequences. If left untreated, a UCFJD can progress to a bilateral cervical facet joint dislocation (BCFJD) [4], which is an unstable cervical spine injury. Owing to the serious implications of a missed UCFJD, it is imperative to perform computed tomography scans whenever there is clinical suspicion of a cervical spine injury, even when initial plain radiographs may not suggest any abnormality [5].

Given the complexity of cervical facet joint dislocations, radiologists rely on metaphoric imaging signs to identify these injuries [6]. In this review, we highlight some of the classical radiological signs which can facilitate the diagnosis of cervical facet joint dislocations.

### Mechanism of Injury

The stability of the cervical spine is mainly contributed by its supporting ligaments [7]. Ligamentous injuries in facet joint dislocation often result from flexion-distraction (primarily hyperflexion) injuries with a rotational component about an axis anterior to the vertebral body [8,9]. The inferior articular facet of the upper vertebra projects anterior to the superior articular facet of the lower vertebra and becomes locked, giving rise to UCFJD [9]. Likewise, when both facet joints are involved and locked, it results in BCFJD [10].

### Bow-Tie Sign and Laminar Space Signs (***Figure 1***)

These signs are associated with UCFJD and best seen on plain radiographs. On the lateral radiograph, one can observe up to 25% translation of the superior vertebral body onto the anteroposterior diameter of the inferior vertebral body [12].

**Figure 1:**
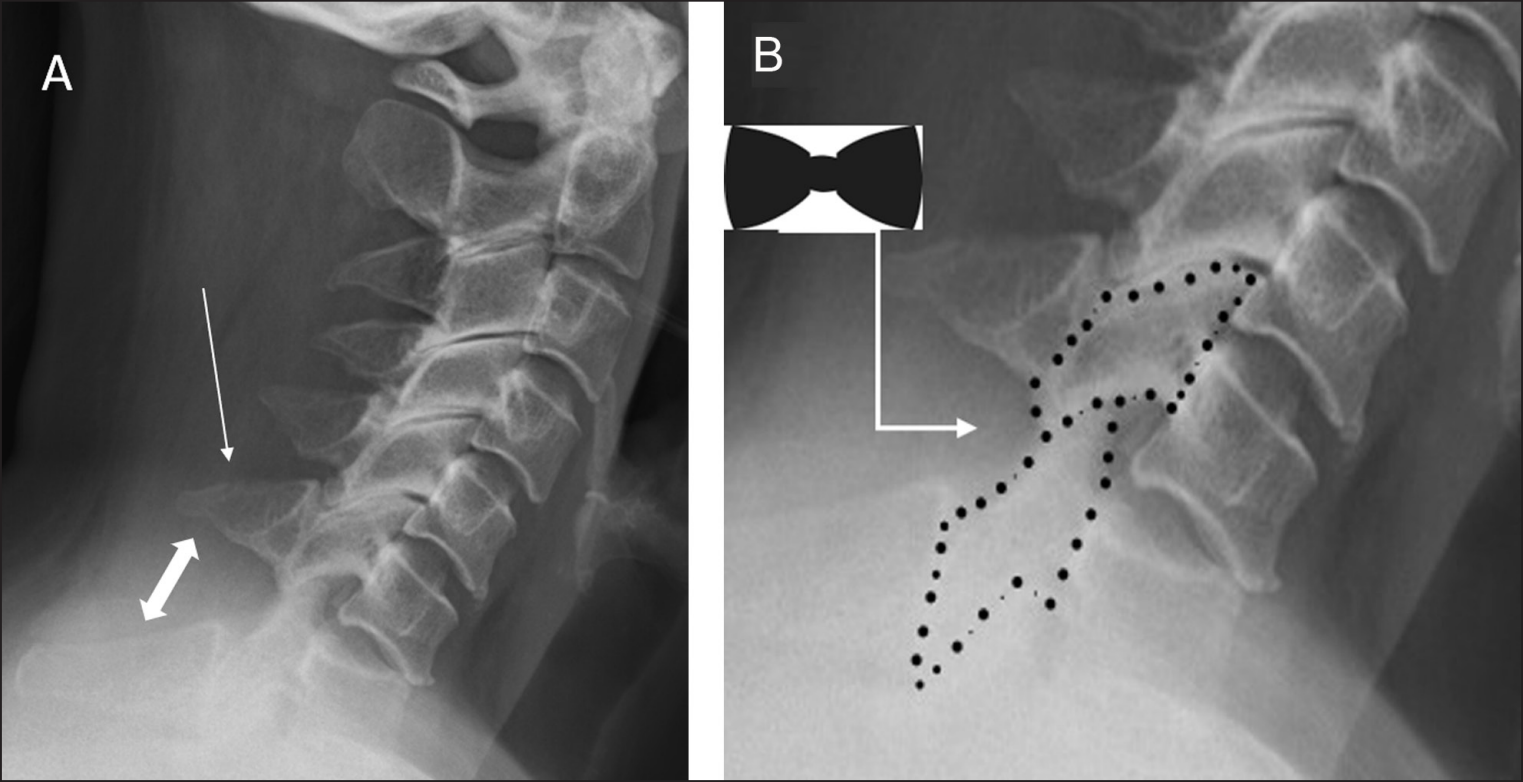
Bow-tie sign and laminar space sign. Lateral radiograph of the cervical spine (A) of a 33-year-old man following a fall from an electric scooter shows a grade I anterolisthesis with less than 25% anterior translation of C6 on C7 vertebrae. In addition, there is an oblique fracture across the spinous process of C6 vertebra, which is consistent with clay-shoveler fracture (single-headed arrow). Besides focal kyphosis and reduced overlap of the articular processes, there is fanning or widening of the interspinous distance (double-headed arrow). The laminar space (which is the distance between the spinolaminar line and the posterior surface of the articular pillars) widens progressively from C3 to C6 level. It changes abruptly and narrows at the C7 level (B). These changes indicate a sudden rotational injury at C6–7 level. Displacement of the interfacetal joint with rotational deformity at this level produces a “bow-tie” appearance and a “double-appearance” of articular facets above the C6–7 level.

The laminar space—which is the distance between the spinolaminar line and the posterior surface of the articular pillars—changes abruptly at the level of the injury due to sudden rotational forces from the injury [11]. This abrupt change in the laminar space is referred to as the “laminar space sign”. Displacement of the interfacetal joint with rotational deformity produces a “bow-tie” appearance at the level of injury; hence it is referred to as the “bow-tie sign” [11]. In addition, this produces a “double-appearance” of articular facets above the level of the injury.

### Stages of Facet Joint Dislocation

According to Allen and Ferguson, the traumatic cervical facet joint dislocation can be summarized into four stages [12]:

Stage I: Subluxation of the facet with increased gap distance between interspinous ligaments. This indicates compromise to the integrity of the posterior ligamentous complex.

Stage II: UCFJDs, with up to 25% translation of the superior vertebral body onto the anteroposterior diameter of the inferior body.

Stage III: BCFJDs, with up to 50% translation of the superior vertebral body onto the anteroposterior diameter of the inferior body.

**Figure 2:**
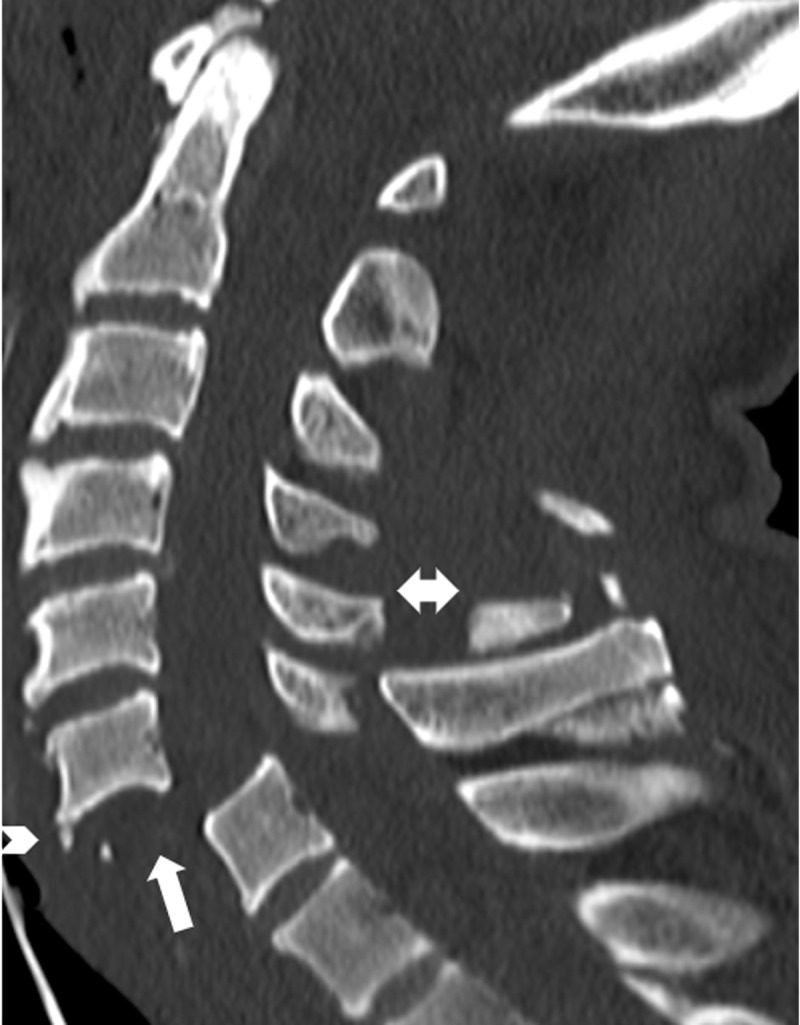
Floating vertebra sign and teardrop sign. Sagittal CT cervical spine of a 77-year-old man following a high energy motor vehicle accident shows 100% translation of the C6 on C7 vertebral bodies, which is consistent with a grade IV anterolisthesis. The appearance of a “floating vertebra” (upward pointing arrow) is associated with bilateral facet joint dislocation and injury to the posterior ligamentous complex. In addition, there are fractures involving the C5 and C6 spinous processes (double head arrow). The mechanism of injury is secondary to forceful muscle contraction, transmitted through the supraspinous ligaments. The tremendous contraction force produces the avulsion fractures of the spinous process known as clay-shoveler fracture. Note the presence of a tiny anterior “teardrop” fracture at the anterior and inferior aspect of the C6 vertebral body (arrow head).

Stage IV: Nearly 100% translation of the superior vertebral body onto the inferior body, resulting in the appearance of a “floating vertebra” (***Figure 2***).

### Hamburger Bun Sign, Naked Facet Sign and Reverse Hamburger Bun Signs (***Figure 3***)

Healthy facet joints appear as “hamburgers” on axial CT scans of the cervical spine, where each facet represents half of the “hamburger bun”. Facet joint dislocations upset this relationship and reverse the orientation of the “half buns”, giving rise to the “reverse hamburger bun sign” [10].

**Figure 3:**
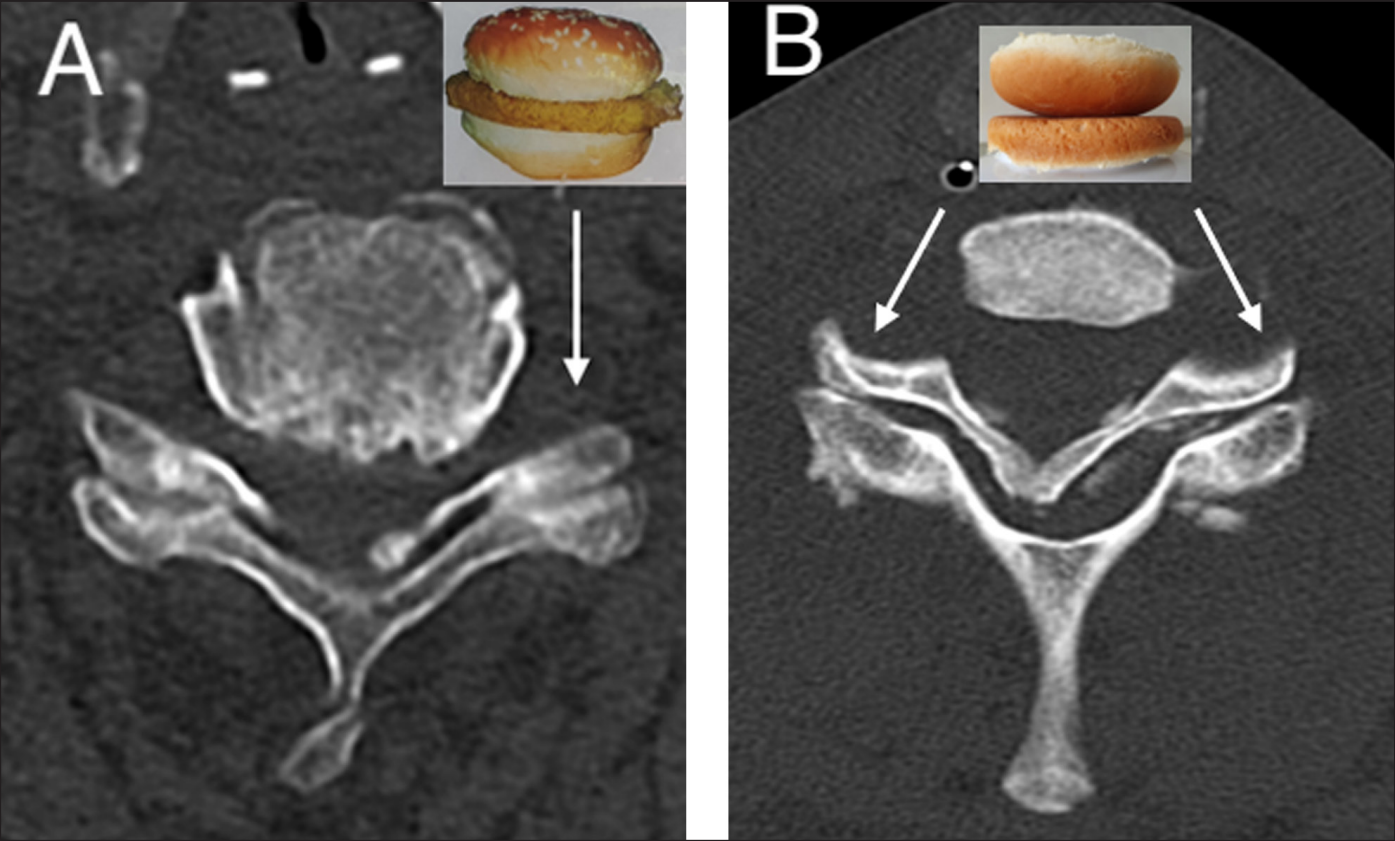
Hamburger bun sign, reverse hamburger bun sign and Naked facet sign. The “hamburger bun” sign resembles the normal appearance of facet joints on axial CT cervical spine scan of the before-mentioned 77-year-old man (A). Facet joint dislocations upset this relationship and reverse the orientation of the “bun” halves to each other. Hence, the “reverse hamburger bun sign”, also known as “naked facet sign”, represents the appearance on axial CT scan of an exposed facet joint at C6–7 level when it is dislocated (B). These signs are often seen in either unilateral or bilateral facet joint dislocation as a result of flexion-distraction injury—where the inferior articular facet of the upper vertebra projects anterior to the superior articular facet of the inferior vertebra.

This is also known as the “naked facet sign”, which represents the appearance of an exposed facet joint after dislocation [10,13].

### Headphones Sign (***Figure 4***)

The headphones sign is best appreciated on the axial CT scan. The concentric appearance of bilateral uncovertebral joints in a healthy vertebra resembles a pair of headphones on its wearer’s head [14]. In a fracture or dislocation of the uncovertebral joint, the superior vertebral body is no longer supported by the uncinate process of the inferior vertebra. Thus, the superior vertebral body is translated anterior to the inferior vertebra, and it rotates towards the normal side. This appearance gives rise to the unilateral positive “headphones sign”, which is commonly associated with UCFJD [14]. On axial CT scans, the “headphones sign” and “reverse hamburger bun sign” are reliable indicators of cervical facet joint dislocations.

**Figure 4:**
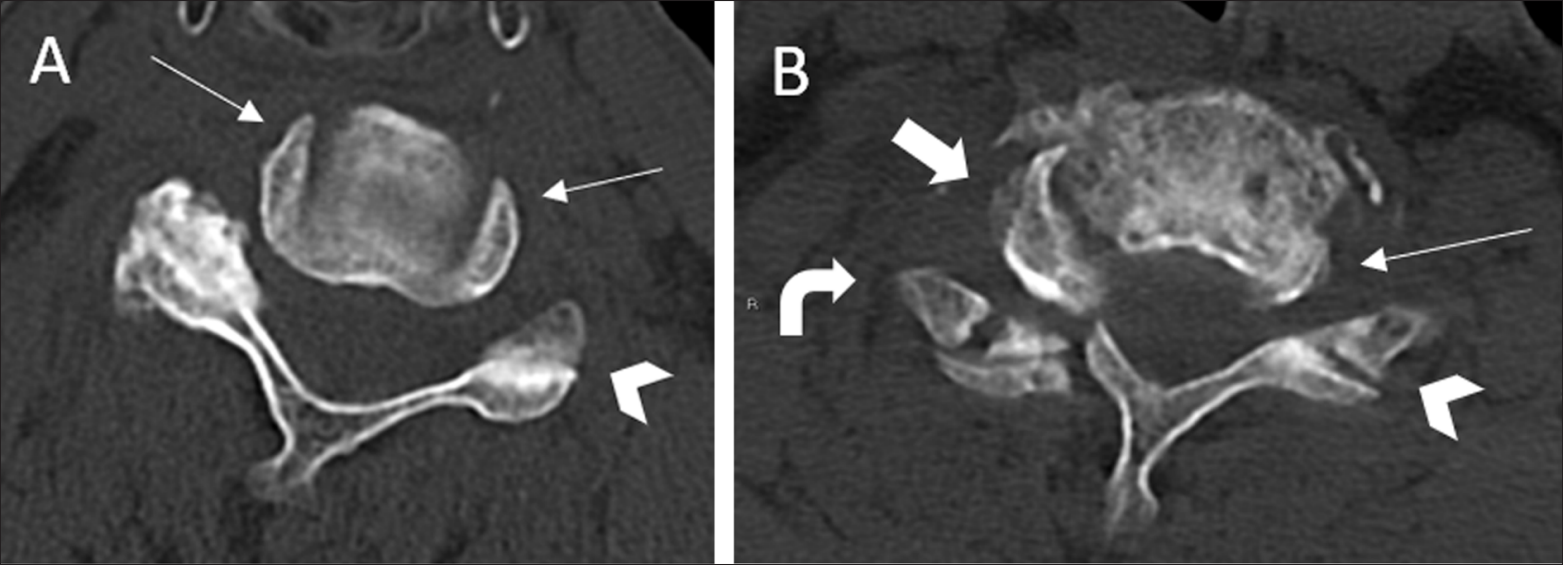
Headphones sign. Axial CT image of a cervical vertebra (A) shows normal concentric appearance of bilateral uncovertebral joints (arrows). This resembles the appearance of headphones on the wearer’s head. Axial CT image of the cervical vertebra (B) of an 80-year-old man following a fall accident shows a dislocated right uncovertebral joint (thick arrow). The vertebral body is no longer framed by the uncinate process of the lower vertebra; instead, it has moved anteriorly and is rotated towards the normal left side. This appearance gives rise to a unilateral positive “headphones sign” (thick arrow), which is commonly associated with unilateral cervical facet joint dislocation (UCFJD). In addition, the normal left facet joint resembles the appearance of hamburger buns (B, arrow head) while the dislocated right facet joint resembles a reverse hamburger bun (B, curved arrow), giving rise to the reverse “hamburger bun sign”. Hence, the positive “headphones sign” and reverse “hamburger bun sign” are both reliable indicators of cervical facet joint dislocation on axial CT imaging.

## Conclusion

The classic radiological signs seen in patients with traumatic cervical facet joint dislocation are inspired by nature. These signs correlate with the anatomical abnormalities presented in cervical spine injuries, thus facilitating the diagnosis of the injuries. Trainees and radiologists may both benefit from this pattern-based approach of learning and practising radiology. Additionally, this pattern recognition approach of learning radiology may be useful in future machine-based learning applications.

## Highlights

1. Classical radiological signs in traumatic cervical facet joint dislocations.
2. “Bow-tie sign” and “laminar space signs” represents UCFJD on plain radiographs.
3. On axial CT, unilateral “reversed hamburger sign”, “naked facet sign” and “headphones sign” represents UCFJD. Likewise, if these signs are present bilaterally, it indicates BCFJD.
